# Mean Corpuscular Volume as a Prognostic Factor for Patients With Habitual Alcohol or Tobacco Use After Esophagectomy

**DOI:** 10.3389/fonc.2021.752229

**Published:** 2021-11-16

**Authors:** Shu-jie Huang, Peng-fei Zhan, Shao-bin Chen

**Affiliations:** ^1^ Department of Clinical Laboratory, Shantou Hospital Traditional Chinese Medicine, Shantou, China; ^2^ Department of Thoracic Surgery, Cancer Hospital of Shantou University Medical College, Shantou, China

**Keywords:** esophageal neoplasm, mean corpuscular volume, prognosis, squamous cell carcinoma, surgery

## Abstract

**Background:**

The goal of this study was to investigate the impact of mean corpuscular volume (MCV) in patients with esophageal squamous cell carcinoma (ESCC) who underwent surgical resection.

**Methods:**

A total of 615 patients with ESCC who underwent esophagectomy were analyzed. Patients were divided into two groups according to the standard MCV: the high MCV group (>100 fl) and the low MCV group (≤100 fl). Survival analyses were performed to calculate overall survival (OS) and cancer-specific survival (CSS) and investigate the independent prognostic factors.

**Results:**

Fifty-one patients (8.3%) were in the high MCV group, and the other 564 patients (91.7%) were defined as the low MCV group. MCV was significantly correlated with sex, habitual alcohol or tobacco use, tumor length, body mass index, and multiple primary malignancies (P < 0.05). Elevated MCV was significantly correlated with poor survival in univariate and multivariate analyses. However, in subgroup analyses, MCV was found to be correlated with survival only in patients with alcohol or tobacco consumption and not in patients without alcohol or tobacco consumption.

**Conclusions:**

Pretreatment MCV was correlated with survival in ESCC patients after esophagectomy. However, its prognostic value might only exist in patients with alcohol or tobacco consumption.

## Introduction

Esophageal carcinoma is a common digestive system malignancy with high mortality (1). Esophagectomy remains the most important tool for treatment in resectable cases, while neoadjuvant chemoradiotherapy is recommended for locally advanced diseases (2). The identification of factors associated with high risk prior to treatment is important for planning the individual therapeutic strategy for patients with malignancies. Currently, the TNM staging system is widely used for predicting the outcomes of esophageal cancer and other malignancies. Although a separate clinical stage (cTNM) was provided in the 8th edition for ESCC to be used as a prognostic indicator before treatment, its predictive value is still limited. Therefore, we think that it is necessary to develop other easily accessible and effective indicators to predict the outcome of esophageal cancer patients before treatment, which may help to improve individualize their treatment.

Mean corpuscular volume (MCV) is a measure of the average volume of erythrocytes. Elevated MCV is always associated with deficiency of folate and vitamin B12, which may be seen in patients with a history of gastrectomy, malnutrition due to alcohol abuse, and so on (3). Recently, MCV was found to be correlated with the survival of patients with several malignancies, such as liver cancer, gastroesophageal adenocarcinomas, colorectal cancer, and head and neck cancer (3–8).

Alcohol and tobacco abuse have been recognized as risk factors for various cancers. Previous studies also showed that elevated MCV was more often found in patients with high alcohol or tobacco consumption (9, 10). To the best of our knowledge, only two studies have evaluated the predictive significance of MCV in patients with esophageal cancer who have undergone surgical resection (11, 12). However, neither of these studies evaluated the prognostic value of MCV according to the status of alcohol and tobacco consumption.

In this study, we investigated the prognostic value of MCV in patients with esophageal squamous cell carcinoma (ESCC) who underwent surgical resection and tried to elucidate the prognostic significance of MCV in these patients according to the history of alcohol and tobacco consumption.

## Patients and Methods

### Patients

Between September 2014 and December 2017, 817 patients with esophageal cancer underwent esophagectomy at Shantou University Medical College Cancer Hospital. Only patients with ESCC who underwent surgery as their initial treatment were included in this study. Patients with a past history of gastrectomy were also excluded from this study. This study was approved by the Ethics Committee of our hospital. Written informed consent was signed for all patients.

### Data Collection

All clinicopathological data and laboratory data were obtained from the patients’ medical records. The stage of the tumor was classified based on the 8th edition American Joint Committee on Cancer TNM staging system for ESCC. Habitual alcohol use was defined as drinking ≥1 time per week, and habitual tobacco use was defined as smoking at least 1 cigarette per day for at least one year. Weight, height, and laboratory data were collected within 1 week before surgery.

The cutoff values of MCV and serum albumin were determined according to the standard value in our hospital. Patients were divided into a high MCV group (>100 fl) and a low MCV group (≤100 fl) as well as a high serum albumin group (≥40 g/L) and a low serum albumin group (<40 g/L). Anemia was defined as Hb < 12 g/dL for women and Hb <13 g/dL for men according to the World Health Organization guidelines (13).

### Surgery

Most of the patients underwent esophagectomy through a right thoracotomy, while other patients underwent a left thoracotomy. For lymphadenectomy, the regional lymph nodes in the middle mediastinal, lower mediastinal, and upper abdominal regions were routinely dissected in all patients. For patients who underwent esophagectomy through a right thoracotomy, the lymph nodes around the left and right recurrent laryngeal nerves were also dissected. Postoperative morbidity was defined as a state where the Clavien–Dindo classification was II or higher (14).

### Statistical Analyses

The smoking index was calculated by daily cigarette intake × number of years smoking. The alcohol index was calculated as daily ethanol intake (g) × number of years drinking. Continuous variables were compared using Student’s t-test. Categorical variables were compared by the χ2 test or Fisher’s exact test. Overall survival (OS) time was calculated from the date of operation to the date of death or most recent follow-up. Cancer-specific survival (CSS) was defined as survival from the date of operation until death due to esophageal carcinoma in the absence of other causes. Survival was calculated using the Kaplan-Meier method, and the differences in survival between groups were compared by the log-rank test. Multivariate Cox regression analyses were applied to identify independent prognostic factors. P< 0.05 was set as significant. All statistical analyses were conducted in SPSS 20.0 software (IBM, Armonk, New York, USA).

## Results

### Patient Characteristics

Of the 817 patients with esophageal carcinoma who underwent esophagectomy between September 2014 and December 2017, 761 patients were diagnosed with ESCC. One hundred sixteen patients who received neoadjuvant therapy were excluded from this study (including 94 patients with neoadjuvant chemoradiotherapy, 13 patients with neoadjuvant radiotherapy, and 9 patients with neoadjuvant chemotherapy). Five patients with a past history of gastrectomy and 25 patients lacking any follow-up data were also excluded. Thus, 615 patients were enrolled for analysis in this study. There were 472 men and 143 women, and the median age was 61 years (range, 38 to 84 years). The mean number of lymph nodes dissected was 26.9 ± 11.0, and the median number was 26 (range, 6-74). Based on the 8th edition of the TNM staging system, 283 patients (46.0%) had pN0 disease, 206 patients (33.5%) had pN1 disease, 99 patients (16.1%) had pN2 disease, and 27 patients (4.4%) had pN3 disease. Radical resection was achieved in 590 patients (95.9%), while palliative resection was performed in 25 patients (4.1%). The postoperative morbidity rate was 8.3% (51/615). The hospital mortality rate was 0.5% (3/615).

One hundred and sixty-eight patients received postoperative adjuvant therapy, including 64 cases of postoperative chemotherapy, 79 cases of postoperative radiotherapy, and 25 cases of postoperative chemoradiotherapy. The most common regimen for chemotherapy was Paclitaxel + Cisplatin or 5-Fu + Cisplatin. A total dose of 40–66 Gy (median 50Gy) irradiation (2 Gy/day, 5 days per week) was administered for postoperative therapy.

Two hundred twenty-five patients habitually drank alcohol, while the other 390 patients did not. The mean alcohol index for these 225 patients with habitual alcohol consumption was 3344.3 ± 117.6. Four hundred thirty-three patients had habitual tobacco use, while the other 182 patients did not. The mean smoking index for these 433 patients with habitual tobacco consumption was 932.5 ± 22.2. Twenty-two patients had multiple primary malignancies (including 5 patients with synchronous malignancy and 17 patients with metachronous malignancy). One hundred twenty-five patients were in the low serum albumin group, while the other 490 patients were defined as the high serum albumin group. Fifty-one patients (8.3%) were in the high MCV group, and the other 564 patients (91.7%) were in the low MCV group.

### Correlation Between MCV and Clinicopathological Factors

**Table 1** shows patient clinicopathological factors stratified by MCV. MCV was significantly correlated with sex, habitual alcohol or tobacco use, tumor length, body mass index (BMI), and multiple primary malignancies (P < 0.05). None of the female patients (0/143) had high MCV in this study, compared with 10.8% of the male patients (51/472) with high MCV (P<0.001). A total of 11.3% of the patients with habitual tobacco use had high MCV, which was significantly higher than the 1.1% of patients without habitual tobacco use (P<0.001). The incidence of elevated MCV was higher in patients with habitual alcohol use than in patients without habitual alcohol use (18.2% *vs* 2.6%, P<0.001). Higher MCV was also more often found in patients with longer tumor length, lower BMI, and multiple primary malignancies.

**Table 1:** Correlation of the mean corpuscular volume with the clinicopathological features.

	No. Patients	MCV		X^2^	*P* value
		≤100fl	>100fl		
Sex				16.848	<0.001
Male	472	421 (89.2%)	51 (10.8%)		
Female	143	143 (100%)	0 (0%)		
Age (yr)				0.078	0.780
≤60	302	276 (91.7%)	26 (8.3%)		
>60	313	288 (92.0%)	25 (8.0%)		
Tumor location				0.549	0.760
Upper third	110	99 (90.0%)	11 (10.0%)		
Middle third	385	355 (92.2%)	30 (7.8%)		
Lower third	120	110 (91.7%)	10 (8.3%)		
Anemia				0.165	0.685
Yes	131	119 (90.8%)	12 (9.2%)		
No	484	445 (91.9%)	39 (8.1%)		
Habitual tobacco use				17.590	<0.001
Yes	433	384 (88.7%)	49 (11.3%)		
No	182	180 (98.9%)	2 (1.1%)		
Habitual alcohol use				45.999	<0.001
Yes	225	184 (81.8%)	41 (18.2%)		
No	390	380 (97.4%)	10 (2.6%)		
Tumor length				4.507	0.034
≤5cm	430	401 (93.3%)	29 (6.7%)		
>5cm	185	163 (88.1%)	22 (11.9%)		
Histologic grade				1.285	0.526
Well	210	196 (93.3%)	14 (6.7%)		
Moderate	317	289 (91.2%)	28 (8.8%)		
Poor	88	79 (89.8%)	9 (10.2%)		
BMI				7.882	0.019
≤18.5	116	100 (86.2%)	16 (13.8%)		
18.5-24.9	444	410 (92.3%)	34 (7.7%)		
≥25.0	55	54 (98.2%)	1 (1.8%)		
Serum albumin				2.835	0.092
<40g/L	125	110 (88.0%)	15 (12.0%)		
≥40g/L	490	454 (92.7%)	36 (7.3%)		
Thoracotomy				<0.001	0.983
Left	156	143 (91.7%)	13 (8.3%)		
Right	459	421 (91.7%)	38 (8.3%)		
Resection margin				0.003	0.957
Radical	590	541 (91.7%)	49 (8.3%)		
Palliative	25	23 (92.0%)	2 (8.0%)		
Postoperative morbidity				0.167	0.683
Yes	51	46 (90.2%)	5 (9.8%)		
No	564	518 (91.8%)	46 (8.2%)		
pT category				6.423	0.093
pT1	73	69 (94.5%)	4 (5.5%)		
pT2	101	97 (96.0%)	4 (4.0%)		
pT3	369	336 (91.1%)	33 (8.9%)		
pT4	72	62 (86.1%)	10 (13.9%)		
pN category				5.419	0.144
pN0	283	265 (93.6%)	18 (6.4%)		
pN1	206	187 (90.8%)	19 (9.2%)		
pN2	99	90 (90.9%)	9 (9.1%)		
pN3	27	22 (81.5%)	5 (18.5%)		
pTNM stage				5.258	0.154
I	75	72 (96.0%)	3 (4.0%)		
II	150	140 (93.3%)	10 (6.7%)		
III	308	281 (91.2%)	27 (8.8%)		
IVA	82	71 (86.6%)	11 (13.4%)		
Multiple primary malignancies				10.808	0.001
Yes	22	16 (72.7%)	6 (27.3%)		
No	593	548 (92.4%)	45 (7.6%)		
Adjuvant therapy					
Yes	168	146 (89.0%)	18 (11.0%)	2.117	0.146
No	447	418 (92.7%)	33 (7.3%)		

BMI, body mass index; MCV, mean corpuscular volume.

### Survival and Prognostic Factors

The last follow-up was conducted in December 2020, after a mean follow-up time of 34.7 months (range, 1-69 months). Two hundred thirteen patients died, and 10 patients were lost to follow-up (1.6%).

**Figure 1:**
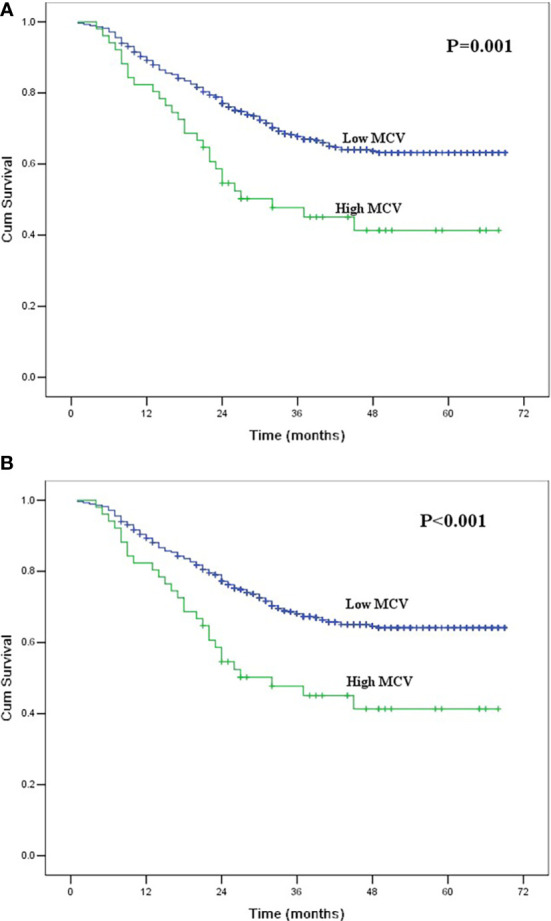
**(A)** Kaplan-Meier curves for overall survival of the entire group according to mean corpuscular volume. The survival difference was significant (P = 0.001). **(B)** Kaplan-Meier curves for cancer-specific survival of the entire group according to mean corpuscular volume. The survival difference was significant (P < 0.001).

The 1-, 3- and 5-year OS rates for all patients were 88.6%, 66.0% and 61.3%, respectively. The 1-, 3- and 5-year CSS rates were 88.8%, 66.4% and 62.2%, respectively. The correlations between the clinicopathological factors and survival are shown in **Table 2**. In univariate analysis, the variables correlated with OS and CSS were tumor length, thoracotomy, resection margin, pT category, pN category, pTNM stage, multiple primary malignancies, and MCV. The 5-year OS and CSS for patients in the low MCV group were 63.2% and 64.1%, respectively, which were significantly higher than the 41.3% and 41.3% for patients in the high MCV group (P<0.05, **Figure 1**). Other factors correlated with poor survival were longer tumor length, lower serum albumin, left thoracotomy, palliative resection, advanced pT category, advanced pN category, advanced pTNM stage, and multiple primary malignancies.

**Table 2:** Univariate analysis in regard to overall survival and cancer-specific survival according to clinicopathological factors.

Variable	5-yr OS (%)	*P* value	5-yr CSS (%)	*P* value
Sex		0.130		0.126
Male	59.4		60.2	
Female	67.6		68.8	
Age (yr)		0.982		0.760
≤60	61.3		61.3	
>60	61.4		63.2	
Tumor location		0.514		0.555
Upper third	60.5		60.5	
Middle third	62.5		63.5	
Lower third	56.7		58.1	
Anemia		0.595		0.599
Yes	60.8		61.9	
No	61.5		62.3	
Habitual tobacco use		0.341		0.357
Yes	59.6		60.4	
No	65.3		66.2	
Habitual alcohol use		0.091		0.104
Yes	57.2		58.3	
No	63.7		64.5	
Tumor length		<0.001		<0.001
≤5cm	66.4		66.6	
>5cm	49.8		51.9	
Histologic grade		0.292		0.245
Well	64.4		66.0	
Moderate	59.0		59.7	
Poor	63.4		63.4	
BMI		0.105		0.130
≤18.5	54.5		57.0	
18.5-24.9	62.1		62.5	
≥25.0	70.1		71.5	
Serum albumin		0.013		0.008
<40g/L	51.8		51.8	
≥40g/L	63.8		64.9	
Thoracotomy		0.005		0.004
Left thoracotomy	53.5		54.5	
Right thoracotomy	64.6		64.8	
Resection margin		<0.001		<0.001
Radical	62.8		63.7	
Palliative	22.7		22.7	
Postoperative morbidity		0.448		0.559
Yes	58.0		60.5	
No	61.6		62.3	
pT category		<0.001		<0.001
pT1	88.2		88.2	
pT2	64.4		65.6	
pT3	60.9		61.5	
pT4	33.2		35.3	
pN category		<0.001		
pN0	75.8		77.7	
pN1	57.6		57.6	
pN2	39.7		39.7	
pN3	21.2		21.2	
pTNM stage		<0.001		<0.001
I	87.6		89.2	
II	74.6		75.2	
III	57.2		58.4	
IVA	28.5		28.5	
Multiple primary malignancies		0.004		0.003
Yes	26.8		26.8	
No	62.7		63.6	
Adjuvant therapy		0.189		0.165
Yes	63.1		63.9	
No	60.6		61.4	
MCV		0.001		<0.001
≤100fl	63.2		64.1	
>100fl	41.3		41.3	

BMI, body mass index; CSS, cancer-specific survival; MCV, mean corpuscular volume; OS, overall survival.

The multivariate analysis incorporated factors that were significant in the univariate analyses (**Table 3**). Thoracotomy, resection margin, pT category, pN category, and MCV were independent prognostic factors for OS and CSS in this study. However, tumor length, serum albumin, pTNM stage, and multiple primary malignancies were not independent risk factors.

**Table 3:** Multivariate analysis in regard to overall survival and cancer-specific survival of the 615 patients with esophageal squamous cell carcinoma.

Prognostic factor	Hazard ratio	95%CI	*P* value
Overall survival			
Tumor length	1.120	0.833-1.507	0.452
Serum albumin	0.875	0.638-1.200	0.407
Thoracotomy	0.555	0.414-0.743	<0.001
Resection margin	2.904	1.635-5.157	<0.001
pT category	1.511	1.130-2.020	0.005
pN category	1.821	1.437-2.308	<0.001
pTNM stage	0.890	0.621-1.274	0.523
Multiple primary malignancies	1.306	0.723-2.360	0.377
MCV	1.546	1.042-2.318	0.032
Cancer-specific survival			
Tumor length	1.068	0.791-1.441	0.668
Serum albumin	0.850	0.619-1.167	0.314
Thoracotomy	0.547	0.407-0.735	<0.001
Resection margin	2.977	1.673-5.296	<0.001
pT category	1.477	1.101-1.981	0.009
pN category	1.845	1.453-2.342	<0.001
pTNM stage	0.915	0.637-1.315	0.632
Multiple primary malignancies	1.298	0.717-2.349	0.389
MCV	1.569	1.056-2.358	0.019

CI, confidence interval; MCV, mean corpuscular volume.

### Prognostic Value of MCV According to Alcohol and Tobacco Consumption

**Figure 2:**
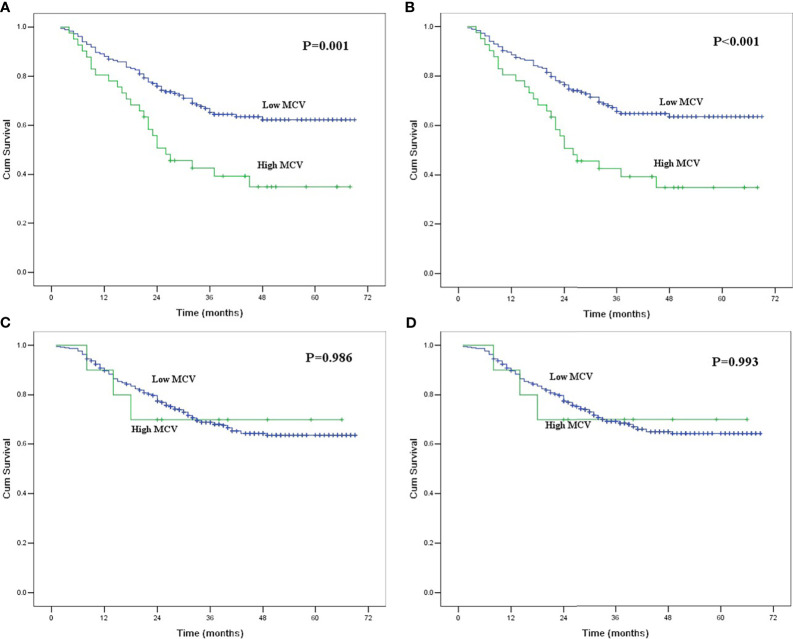
**(A)** Kaplan-Meier curves for overall survival of the patients with habitual alcohol use according to mean corpuscular volume. The survival difference was significant (P = 0.001). **(B)** Kaplan-Meier curves for cancer-specific survival of the patients with habitual alcohol use according to mean corpuscular volume. The survival difference was significant (P < 0.001). **(C)** Kaplan-Meier curves for overall survival of the patients without habitual alcohol use according to mean corpuscular volume. The survival difference was not significant (P = 0.986). **(D)** Kaplan-Meier curves for cancer-specific survival of the patients without habitual alcohol use according to mean corpuscular volume. The survival difference was not significant (P = 0.993).

We further conducted subgroup analyses to identify the value of MCV according to the history of alcohol and tobacco consumption. Of 225 patients with habitual alcohol use, 41 patients had high MCV, and 184 patients had low MCV. The mean alcohol index for patients with high MCV was 4213.4 ± 426.1, which was higher than the 3150.7 ± 180.4 for patients with low MCV (P = 0.016). High MCV was found to be significantly correlated with poor OS and CSS in ESCC patients with habitual alcohol use (P<0.05, **Figure 2**). However, MCV was not correlated with survival in the 390 patients without habitual alcohol use (P>0.05, **Figure 2**).

**Figure 3:**
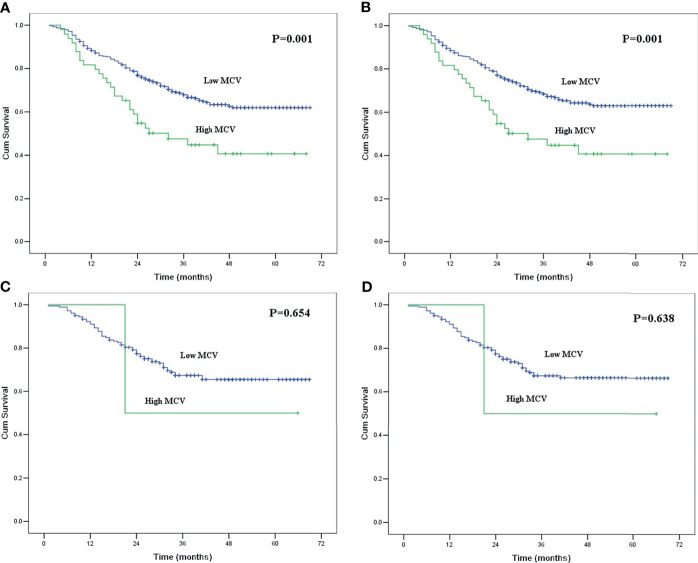
**(A)** Kaplan-Meier curves for overall survival of the patients with habitual tobacco use according to mean corpuscular volume. The survival difference was significant (P = 0.001). **(B)** Kaplan-Meier curves for cancer-specific survival of the patients with habitual tobacco use according to mean corpuscular volume. The survival difference was significant (P = 0.001). **(C)** Kaplan-Meier curves for overall survival of the patients without habitual tobacco use according to mean corpuscular volume. The survival difference was not significant (P = 0.654). **(D)** Kaplan-Meier curves for cancer-specific survival of the patients without habitual tobacco use according to mean corpuscular volume. The survival difference was not significant (P = 0.638).

Of the 433 patients with habitual tobacco use, 49 patients had high MCV, and 384 patients had low MCV. The mean smoking index for patients with high MCV was 1079.6 ± 71.4, which was higher than the 913.7 ± 23.2 for patients with low MCV (P = 0.018). In survival analysis, MCV was also found to be correlated with survival in ESCC patients with habitual tobacco use but not in patients without habitual tobacco use (**Figure 3**).

## Discussion

Preoperative malnutrition has been reported to be a predictor of outcomes in cancer patients and can be used as a prognostic indicator (15–17). Recently, MCV has been found to be another nutrition parameter correlated with the survival of various solid malignancies (3–8). Previous studies showed that elevated MCV was always observed in patients with malnutrition due to alcohol abuse (3). Our current study also showed that MCV was significantly correlated with habitual alcohol or tobacco use in patients with ESCC. Moreover, elevated MCV was more often seen in malnutrition patients with low BMI. Surprisingly, none of the 143 female patients in the current study had high MCV, compared with 10.8% among male patients. The explanation for this result may be that few females had habitual alcohol and tobacco abuse in this study. Only 1 female patient with habitual alcohol use and 2 female patients with habitual tobacco use were enrolled in our current study.

To date, only two studies have evaluated the value of MCV in patients with esophageal carcinoma who underwent surgical resection (11, 12), and both of these studies found that elevated MCV was an independent negative prognostic factor. Our current study reported a same result. The 5-year OS and CSS for patients with high MCV were significantly lower than those for patients with low MCV, and MCV was found to be an independent prognostic factor for patients with ESCC after esophagectomy.

Alcohol and tobacco abuse are leading risk factors for death globally and have been found to be correlated with multiple carcinomas, including ESCC (18–21). Elevated MCV has been recognized as a biomarker for habitual alcohol and tobacco abuse (22, 23). Our study also found that elevated MCV was more often seen in patients with alcohol and tobacco consumption, especially in patients with a higher alcohol or smoking index.

Although MCV has been reported to be correlated with the survival of patients with esophageal carcinoma, no studies have investigated the prognostic value of MCV in ESCC patients who had habitual alcohol or tobacco abuse. To the best of our knowledge, our study is the first to demonstrate that MCV was correlated with survival only in ESCC patients with habitual alcohol or tobacco use and not in patients without. Combined with the fact that MCV is more often found in patients with heavy alcohol or tobacco consumption, we can assume that MCV is not only a biomarker for habitual alcohol and tobacco abuse, but also a prognostic factor for ESCC patients with alcohol or tobacco consumption. When we use MCV as a prognostic factor for patients with ESCC, alcohol and tobacco consumption should be taken into account. We think that more studies should be conducted to confirm our findings.

The mechanism by which elevated MCV leads to a poor prognosis of ESCC patients with alcohol or tobacco consumption is still not clear, but there are several possible explanations. First, elevated MCV is always correlated with malnutrition due to a rough lifestyle. In our current study, we found that elevated MCV was more often found in patients with lower BMI, which was an indicator of malnutrition. Previous studies found that malnutrition might be correlated with poor survival in patients with ESCC (24–26). Second, elevated MCV is more often seen in patients with higher alcohol or smoking index. Recent study showed that heavy drinking (>60g per day) represented the largest burden of alcohol-attributable cancer death globally, while esophageal cancer was the most common alcohol-attributable cancer (21). Third, patients with elevated MCV might have a higher incidence of multiple primary malignancies. In our study, six of the 51 patients (11.8%) in the high MCV group developed multiple primary malignancies, while only 2.8% of the patients in the low MCV group developed multiple primary malignancies. A previous study found that the occurrence of multiple primary cancers was associated with poor prognosis in patients with esophageal cancer (27). More studies should be conducted to elucidate the mechanism by which MCV correlates with poor outcomes in ESCC patients.

There are several limitations of this study. First, it was a retrospective study from a single center, which undermined its power. Second, the patient number in some subgroups was too small, which limited the statistical power. For example, there were only two patients with high MCV in the no-habitual-tobacco use group. Third, we did not consider the synergistic effect between alcohol and tobacco in this study, as many patients may consumed both alcohol and tobacco. We think that further studies with larger cohorts are needed to confirm our findings and investigate the mechanism by which MCV relates to the prognosis of ESCC patients.

In conclusion, our study demonstrated that pretreatment MCV was correlated with survival in ESCC patients after esophagectomy. However, its prognostic value might only exist in patients with alcohol or tobacco consumption and not in patients without alcohol or tobacco consumption. Due to the small number of patients with high MCV in the no-habitual-tobacco use group and no-habitual-alcohol use group, we think that a multicenter study with larger cohorts are needed to confirm our findings.

## Data Availability Statement

The original contributions presented in the study are included in the article/supplementary material. Further inquiries can be directed to the corresponding author.

## Ethics Statement

The studies involving human participants were reviewed and approved by the Ethics Committee of Shantou University Medical College Cancer Hospital. The patients/participants provided their written informed consent to participate in this study.

## Author Contributions

S-jH and P-fZ contributed equally to this article. S-bC designed the research, analyzed the data and wrote part of the paper. S-jH and P-fZ analyzed the data and wrote part of the paper. All authors contributed to the article and approved the submitted version.

## Funding

The Medical Scientific Research Foundation of Guangdong Province of China (B2019070).

## Conflict of Interest

The authors declare that the research was conducted in the absence of any commercial or financial relationships that could be construed as a potential conflict of interest.

## Publisher’s Note

All claims expressed in this article are solely those of the authors and do not necessarily represent those of their affiliated organizations, or those of the publisher, the editors and the reviewers. Any product that may be evaluated in this article, or claim that may be made by its manufacturer, is not guaranteed or endorsed by the publisher.
